# A Comparative Evaluation of the Structural and Biomechanical Properties of Food-Grade Biopolymers as Potential Hydrogel Building Blocks

**DOI:** 10.3390/biomedicines10092106

**Published:** 2022-08-28

**Authors:** Adonis Hilal, Anna Florowska, Tomasz Florowski, Małgorzata Wroniak

**Affiliations:** Department of Food Technology and Assessment, Institute of Food Science, Warsaw University of Life Sciences, 02-787 Warsaw, Poland

**Keywords:** protein, polysaccharide, mechanical properties, microrheology, physical stability, gel matrix

## Abstract

The aim of this study was to conduct a comparative assessment of the structural and biomechanical properties of eight selected food-grade biopolymers (pea protein, wheat protein, gellan gum, konjac gum, inulin, maltodextrin, psyllium, and tara gum) as potential hydrogel building blocks. The prepared samples were investigated in terms of the volumetric gelling index, microrheological parameters, physical stability, and color parameters. Pea protein, gellan gum, konjac gum, and psyllium samples had high VGI values (100%), low solid–liquid balance (SLB < 0.5), and high macroscopic viscosity index (MVI) values (53.50, 59.98, 81.58, and 45.62 nm^−2^, respectively) in comparison with the samples prepared using wheat protein, maltodextrin, and tara gum (SLB > 0.5, MVI: 13.58, 0.04, and 0.25 nm^−2^, respectively). Inulin had the highest elasticity index value (31.05 nm^−2^) and MVI value (590.17 nm^−2^). The instability index was the lowest in the case of pea protein, gellan gum, konjac gum, and inulin (below 0.02). The color parameters and whiteness index (WI) of each biopolymer differed significantly from one another. Based on the obtained results, pea protein, gellan gum, konjac gum, and psyllium hydrogels had similar structural and biomechanical properties, while inulin hydrogel had the most diverse properties. Wheat protein, maltodextrin, and tara gum did not form a gel structure.

## 1. Introduction

Hydrogels are a three-dimensional network of hydrophilic polymers with viscoelastic properties. Creating a matrix with high-water content is possible due to the formation of structural networks [1]. Hydrogels have aroused a wide range of interest due to their promising applications as tissue engineering materials [2,3], controlled-release drug delivery carriers [4,5], biomedicine materials [6,7], soft robotic components [8,9], and biosensors [10,11], etc. However, in recent years, the need to address issues such as resource renewability, sustainability, and affordability has been recognized. Furthermore, some applications, particularly in the biomedical [12], food [13], pharmaceutical [14], and cosmetic [15] sectors, require non-synthetic building blocks (polymers).

Naturally sourced hydrogel building blocks (biopolymers) have great potential in achieving the above-mentioned goals, while being biocompatible, biodegradable, and edible. These biopolymers are proteins and polysaccharides with different biomechanical and functional properties [16]. Natural biopolymers are typically obtained from natural resources such as animals (e.g., gelatin [17], whey protein [18], chitosan [19]), plants (e.g., soy protein [20], pea proteins [21], inulin [22], cellulose [23]), microorganisms (e.g., gellan gum [24], curdlan [25]), and algae (e.g., carrageenan [26], agarose [27]) via bioprocessing and chemical modification. Furthermore, because of the high reactivity of their functional groups, these biopolymers can be modified to meet the demands of various specific functions (mechanical properties, solubility, gel structure, etc.) [28]. Although it is a recent trend, plant-based biopolymers are increasingly gaining more interest due to their functional properties and sustainability. Plant polysaccharide-based biopolymers (e.g., gellan gum, konjac gum, inulin, maltodextrin, tara gum, cellulose, starch, pectin) and plant protein-based biopolymers (e.g., soy, pea, zein) are promising hydrogel building blocks [12].

Protein and polysaccharide hydrogels are generally induced by physical (heating, cooling, shear forces, etc.) and chemical (pH modulation, salt addition, etc.) induction techniques, or by a mixed technique to achieve the desired properties [13]. They are mostly formed by physical crosslinking (electrostatic, hydrogen bonding, hydrophobic, or Van der Waals interactions, or their combination). Chemical crosslinking can also occur in the case of the formation of disulfide bonds, the use of enzymes (e.g., glutaminase), or the use of the Millard reaction to induce the cross-linkage of the polymeric chain [1]. When food-grade (safe for human consumption) biopolymers are combined, their gelation and physicochemical properties change. Moreover, combining proteins and polysaccharides can result in a wide variety of structures [29,30]. Such binary food-grade hydrogels can be made by a simple physical complexation, meaning that they can be tuned with variables such as mixing ratio, pH, and ion strength [31]. 

Biopolymer hydrogels have a wide range of potential applications in different sectors. They can be used in tissue engineering to mimic the extracellular matrix (ECM), providing a non-immunogenic biocompatible scaffold [32]. Pre-gel hydrogels are injected, and when applied, they form the appropriate structure that conforms to the shape of the tissue defect. They are considered ideal matrices in the repair of articular cartilage due to the large amount of bound water [33]. Many studies have shown that hydrogels prepared using natural polymers act as an accelerating anti-inflammatory agent in wound healing [34,35]. Hydrogels are highly permeable to various drugs. They are used to release the drug molecule via physical or chemical changes in their structure [36]. For example, these systems are being used as a novel treatment for skin cancer. They are used for transdermal drug delivery, which improves the transport of antitumor agents. However, this treatment system is only applicable to the treatment of skin cancer, as cancerous lesions in other tissues are not readily available to hydrogels. Nevertheless, researchers are attempting to develop hydrogel drug carriers to deliver anti-cancer agents in the treatment of colon, breast, and ovarian cancer [37]. Moreover, natural biopolymer-based hydrogels are being tested for their ability to be used in the production of nontoxic, renewable, wearable, and stretchable biosensors. These hydrogel-based biosensors have a promising potential for detecting physiological parameters, such as body motions and temperature, physical, respiratory rate, humidity, heart rate, and environmental conditions variability. Therefore, they can play crucial roles in everyday human health care [28,38,39]. Because of their ability to deliver and maintain an appropriate therapeutic dose, hydrogels are also widely used in ophthalmic drugs. The high water content and active ingredients of hydrogels increase the effectiveness of treatment, allowing the drug to remain on the surface of the eyeball for up to seven times longer than drugs that do not use hydrogels [40]. Additionally, biopolymeric hydrogels can mimic fat and sugar in various foods. By incorporating the hydrogel into a fat-free (light) product it is possible to maintain the desired creaminess and mouthfeel. This can be achieved by the increasing of mouth surface lubrication, which gives a similar effect to fat [41]. Biopolymeric hydrogels can also be used to control the release of sugars to compensate for the loss of flavor intensity caused by the reduction of sugar and salt in gelled foods [42,43]. Furthermore, the digestion of the denatured proteins used in gel formation is very efficient due to the abundance of peptidases present in the gastrointestinal tract. On the other hand, the digestion of polysaccharides varies extremely. Some types of starch are rapidly hydrolyzed by amylase in the mouth and small intestine, while most other polysaccharides, collectively known as dietary fibers, are not digested but can be fermented by the prebiotic bacteria (e.g., inulin, pectin, psyllium) [44,45]. This can allow the use of biopolymer hydrogels to prolong the satiety effect of foods, and more importantly to enhance the gastrointestinal stability of bioactive compounds they can be carrying [46]. Some applications of biopolymeric hydrogels are highly interdisciplinary (drug delivery system, matrix for tissue culture, etc.). For example, proteins and polysaccharides can be used to produce hydrogels that can deliver bioactive substances, including drugs (pharmaceuticals) to a specific section of the gastrointestinal tract, while protecting the bioactive compound from harsh digestive conditions [47]. These functional bioactive compounds delivery systems can be incorporated into food systems to deliver nutraceuticals. They can also act as therapeutical and smart platforms for carrying pharmaceuticals in many branches of medicine (cardiology, oncology, immunology, and pain management) [2]. Food-grade biopolymers used in the production of hydrogels are safe when used as implantable materials or in cell culture, which is also required in the production of non-mammalian edible scaffolds for in-vitro meat production, which is gaining popularity [48]. Biopolymeric hydrogels are also well known for their biodegradability (related to the possibility of metabolizing into products harmless to humans and the environment) [49]. 

To summarize, it can be stated that food-grade biopolymers (protein and polysaccharides) have a great potential in the biomedical, food, pharmaceutical, and cosmetic sectors due to their renewability, sustainability, affordability, biocompatibility, biodegradability, and edibility. Nonetheless, due to the lack of publications on this topic, preliminary comparative studies of the gelling ability of different biopolymers and their potential as hydrogel building blocks are needed. For this purpose, eight food-grade biopolymers were selected to evaluate their gelling ability and potential as hydrogel building blocks.

## 2. Materials and Methods

### 2.1. Material

Pea protein (PP, NUTRALYS^®^ F85F, protein content 88%, ash 10%), wheat protein (WP, NUTRALYS^®^ W, protein content 85%, ash 10%), and maltodextrin (MD, GLUCIDEX^®^ 1, dextrose equivalent (DE) 5, ash 0.5%) were obtained from Roquette Freres, (Lestrem, France). Gellan gum (GG, high acyl Type 900, particle size: min. 95% mesh through 80 mesh), konjac gum (KG, Type CKHY 1240, particle size: min. 90% through 100–120 mesh), Psyllium Husk Powder (PS, type 10351, purity: 95%, particle size: 60 mesh), and tara gum (TG, Type 5000, particle size: min. 80% through 100 mesh) were obtained from C.E. Roeper GmbH, (Hamburg, Germany). Inulin (INU, Type Orafti^®^ HPX, average degree of polymerization DP ≥ 23) was purchased from BENEO GmbH (Mannhei, Germany).

### 2.2. Samples Preparation

The optimal gelling concentration described in the available literature was considered in determining the concentration level for each biopolymer. Pea protein (PP), wheat protein (WP), inulin (INU), and maltodextrin (MD)—concentration 20 g/100 g. Konjac gum (KG) and psyllium (PS)—concentration 1.5 g/ 100 g. Gellan gum (GG) and tara gum (TG)—concentration 0.4 g/100 g. The preparation of the samples involved dispersing the chosen biopolymers in distilled water (80 °C) using a homogenizer (20,000 rpm for 1 min). Then the solutions were stored (in 20 mL glass vials) for 24 h at a temperature of 8 °C to let them develop a gel-like structure. 

### 2.3. Methods

#### 2.3.1. Volumetric Gelling Index (VGI) and Sample Appearance after Inversion

The VGI was used to assess the degree of hydrogel formation. It is a parameter that expresses the ability of a dispersion to form a gel structure. When the gel structure is not formed, VGI equals zero, and when the sample is completely gelled, VGI equals 100%. VGI is calculated based on the following equation [19]:

$$\mathrm{VGI}=\frac{V_{G}}{V_{T}}\;\times \;100$$

where V_G_—volume of the formulated gel, V_T_—total volume of the sample. The reported values represent the averages of three replicates. Furthermore, the samples were evaluated in terms of their appearance after the vials were inverted. It is a visual test to determine whether a sample has formed a gel structure or is still a sol [50].

#### 2.3.2. Microrheological Properties

The Rheolaser Master device (Formulaction, L’Union, France) was used to investigate the microrheological properties of the samples. The device operates based on dynamic MS-DWS (Multi Speckle Diffusing Wave Spectroscopy) technique in the near-infrared (wavelength of 650 nm). The detector captures the interfering backscattered waves, and the measurement results were recorded using the Rheotest software [51]. Based on the obtained raw data, the following microrheological parameters were determined: Mean Square Displacement (MSD) curves, elasticity index (EI) [nm^−2^], solid–liquid balance (SLB) [nm^2^], and macroscopic viscosity index (MVI) [nm^−2^]. The MSD value is the mean of several scattering trajectories of the particle movement as a function of time in the analyzed sample. EI is directly proportional to the elastic modulus (G’) and is calculated as the reciprocal of the MSD value at the plateau. SLB corresponds to the dimensionless ratio of modulus of elasticity and modulus of viscosity loss G’/G”. MVI is the equivalent of the apparent viscosity at zero shear and is calculated as the reciprocal of the MSD slop [52]. The reported values represent the averages of three replicates.

#### 2.3.3. Physical Stability

The physical stability of the obtained gels was assessed using LUMiSizer 6120-75 (L.U.M. GmbH, Berlin, Germany). This physical stability assessment technic involves subjecting the samples to centrifugal force while illuminating the entire sample cell with near-infrared (NIR) light. The sensor simultaneously measures the intensity of transmitted light as a function of time and position over the entire sample length, and the data is converted and recorded using the provided software (SepView 6.0; LUM, Berlin, Germany). For this analysis, the following parameters were used: dispersion volume 1.8 mL; wavelength 870 nm; light factor 1; 1500 rpm; experiment period 15 h 10 min; interval time 210 s; temperature 20 °C. Based on the recorded data, the destabilization behavior (fingerprint) was obtained, and the instability index was computed [53,54]. The reported values represent the averages of three replicates.

#### 2.3.4. Color Parameters

The color parameters were measured using a CR-5 stationary colorimeter (Konica Minolta, Tokyo, Japan) in the CIE system (L*, a*, b*) with a D65 illuminant. Before each use the device was calibrated, and to exclude the mirror image of the measurement vessel (diameter 5 cm, height 2 cm) in which the sample was placed, the specular component excluded method was used. The measurements were taken five times for each of the three replicates (at a temperature of 20 ± 1 °C). The brightness of the studied sample is indicated by the L* color parameter that ranges from 0 to 100 (higher values means brighter samples). The a* parameter represents the share of green (negative value) and red (positive value) color. The b* color parameter values represent the share of blue (negative value) or yellow (positive value) color in the samples [55]. The reported values represent the averages of three replicates. Additionally, to determine the whiteness of the obtained samples, the whiteness index (WI) of each dispersion was calculated as follows [56]:

$$\mathrm{WI}=100\;-\sqrt{{(100\;-L^{*})}^{2}{+a}^{*2}{+b}^{*2}}$$

where: $L^{*}{,\;a}^{*}{,\;\mathrm{and}\;b}^{*}$ refer to the color parameters of each analyzed sample. To determine the color differences between all the samples, the total color difference parameter ∆E was calculated as follows [55]:

$$\Delta E=\sqrt{{\left(L_{s1}^{*}-L_{s2}^{*}\right)}^{2}+{\left(a_{s1}^{*}-a_{s2}^{*}\right)}^{2}{+(b}_{s1}^{*}-b_{s2}^{*}{)}^{2}}$$

where: $L_{S1}^{*};\;a_{S1}^{*};\;b_{S1}^{*}\;\mathrm{and}\;L_{S2}^{*};\;a_{S2}^{*};\;b_{S2}^{*}$ refer to the color parameters of the compared samples. The color difference between the samples can be estimated as not noticeable for the observer, when 0 < ∆E < 1; only experienced observers can notice the difference, when 1 < ∆E < 2; unexperienced observers can notice the difference, when 2 < ∆E < 3.5; clear color difference is noticed, when 3.5 < ∆E < 5; an observer notices two different colors, when 5 < ∆E.

#### 2.3.5. Statistical Analysis

One-way ANOVA analysis of variance was used to determine the significance of differences between the average values of microrheological parameters (EI, SLB, MVI), the instability index, and the color parameters (L*, a*, b*), and the whiteness index (WI). Tukey’s test at significant level = 0.05 was used to confirm the significant differences between the biopolymers. Additionally, the results were assessed using the principal component analysis (PCA) and hierarchal cluster analysis (HCA). All the analyses were performed using Statistica13.3 (TIBICO Software Inc., Tulsa, OK, USA).

## 3. Results

### 3.1. Volumetric Gelling Index (VGI) and Sample Appearance after Inversion

**Figure 1:**
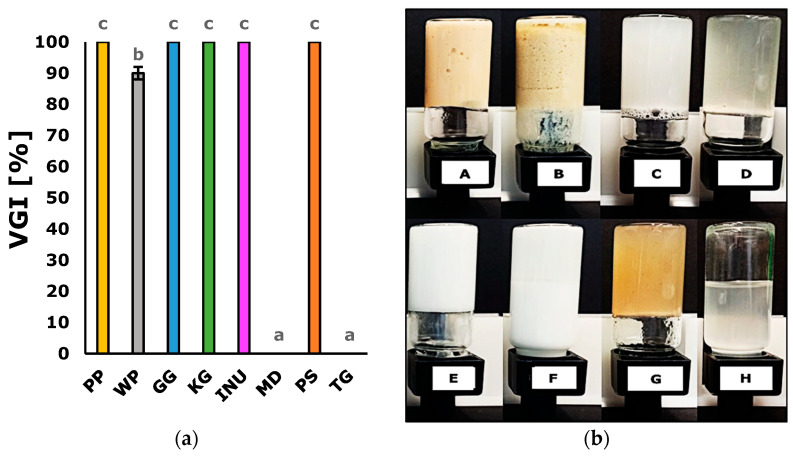
Volumetric gelling index (**a**) of the samples obtained using the analyzed biopolymers, and the appearance of the samples in the vials observed immediately after inversion (**b**), where: (A) pea protein—PP; (B) wheat protein—WP; (C) gellan gum—GG; (D) konjac gum—KG; (E) inulin—INU; (F) maltodextrin—MD; (G) psyllium—PS; (H) tara gum—TG. According to Turkey’s test, the values followed by the same letter (a–c) do not differ significantly (*p* > 0.05).

To determine the ability to form a gel structure by the tested biopolymers the volumetric gelling index (VGI) was determined. The mean values of VGI and the images of the analyzed biopolymers are presented in Figure 1. Based on the visual evaluation, as well as VGI of the obtained samples, it was found that pea protein (PP), gellan gum (GG), konjac gum (KG), inulin (INU), and psyllium (PS) formed a gel-like structure (VGI = 100%). Maltodextrin (MD) and tara gum (TG) were fluid (VGI = 0%). In the case of wheat protein (WP), it was observed that the structure was not homogenous, and some of the aqueous phases got separated (it resembled an insoluble complex that has precipitated, VGI = 90%). It was also observed that GG, KG, and PS were slightly transparent compared to the other samples.

### 3.2. Microrheological Properties

The microrheological properties were determined using the MD-DWS method, which allows for the measurement to be carried out in a non-invasive way (no mechanical/external stress). The rectilinearity of the MSD profiles indicates that the analyzed sample is fluid, exhibiting Newtonian fluid behavior. The non-rectilinear curve path occurs at the transition from fluid to sol. When the examined samples change from sol to gel the MSD profiles achieve a plateau. This means that the particles are not able to move freely due to the formation of a network interaction.

**Figure 2:**
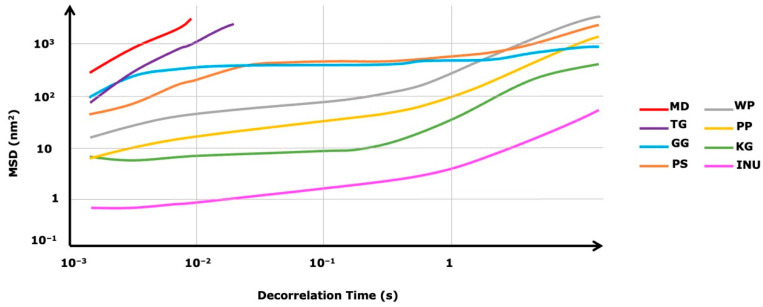
Selected mean square displacement MSD of the analyzed biopolymers, as a function of decorrelation time.

The mean square displacement MSD of each analyzed biopolymer as a function of decorrelation time is presented in Figure 2. Based on the presented MSD profiles, it was observed that in the case of MD and TG the MSD profiles were the most rectilinear, meaning that the particles were freely moving in the continuous phase (fluid/liquid samples). On the other hand, in the case of GG and PS samples, the MDS profiles path was less rectilinear, which means that they were in a sol state (or they were exhibiting properties of a soft gel structure). KG, PP, WP, and INU had non-rectilinear MSD curves that were moving closer to the baseline (INU MSD profile was the closest to the baseline). Additionally, the profiles began to plateau, meaning that the freely moving particles got entrapped in a network structure (cage) that was formed by the viscoelastic system.

The mean values of the microrheology parameters: solid–liquid balance (SLB), elasticity index (EI), and macroscopic viscosity index (MVI) are presented in Table 1. SLB is directly proportional to the viscoelastic properties of the samples and indicates changes in the ratio from liquid-like to solid-like behavior. The samples with the significantly highest SLB value were maltodextrin (MD) and tara gum (TG), which means that they exhibited a more liquid-like behavior—no gel structure (SLB > 0.5). On the contrary, PP, GG, KG, INU, and PS exhibited more solid-like behavior (SLB < 0.5), which can be due to the formation of a gel structure in these samples. In the case of the WP sample, SLB was 0.57 nm^−2^, which means that the sol was close to getting to the gelling point where SLB = 0.5. The elasticity index (EI) is directly proportional to the storage modulus G’ and provides information about the sample’s elasticity, which is due to the solid-like characteristic. Based on the obtained EI values, only INU differed significantly from the rest of the biopolymers, with the highest EI value (31.05 × 10^−2^ nm^−2^). Although the SLB values of PP, GG, KG, and PS showed that they exhibited more solid-like behavior, they had low EI values (comparable to the EI values of MD and TG), which can suggest that their gel structure was softer (less elastic). The MVI value of the inulin (INU) samples reached the highest value, which correlates with the elasticity index (EI). PP, GG, KG, and PS had significantly higher MVI values than those of WP, MD, and TG, which proves that although they all had comparable EI values, the WP, MD, and TG did not form a gel structure.

### 3.3. Physical Stability

**Figure 3:**
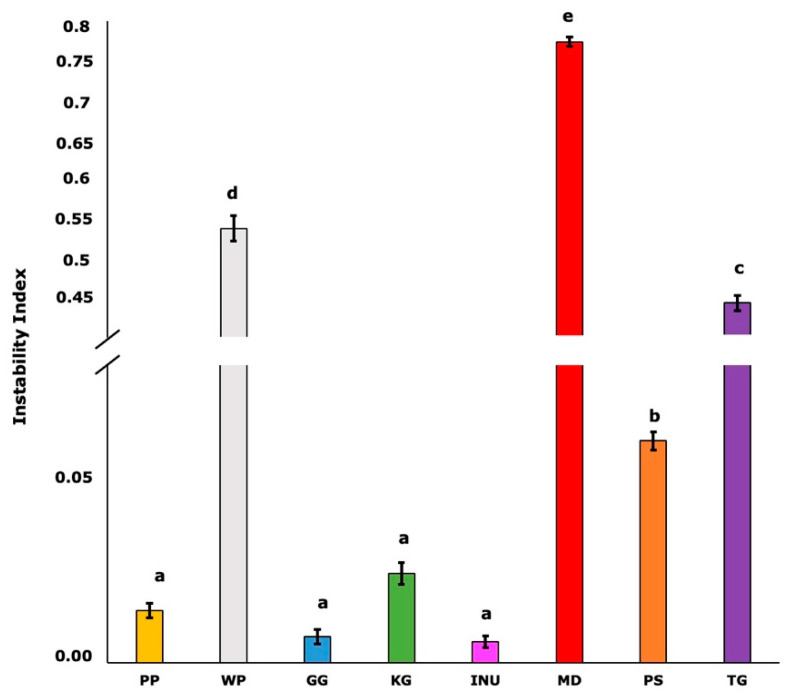
Instability index of the analyzed biopolymers. According to Turkey’s test, the values followed by the same letter (a–e) do not differ significantly (*p* > 0.05).

Physical stability is an important parameter in characterizing the ability of biopolymers to form a hydrogel. To assess the physical stability of the analyzed biopolymers, the instability index was calculated. This parameter ranges from 0 for a stable sample to 1 for an unstable sample. The mean values of the instability index for each biopolymer are presented in Figure 3.

Pea protein (PP), gellan gum (GG), konjac gum (KG), and inulin (INU) samples had the lowest value of the instability index (below 0.02). The instability index of PS was significantly higher than PP, GG, KG, and INU, but still relatively low (0.06). Higher instability indexes were recorded for TG (0.44), WP (0.54), and the most physically unstable were the samples prepared using MD (0.77). 

**Figure 4:**
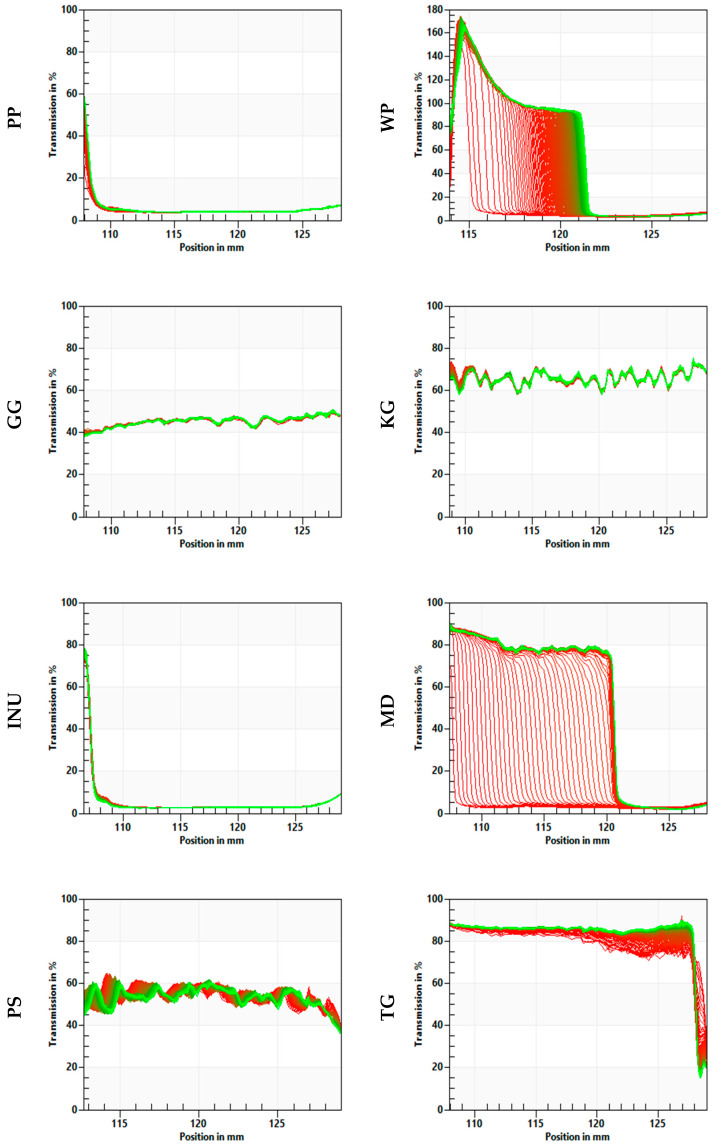
Evolution of transmission profiles (“fingerprints”) of the analyzed biopolymers. The red lines represent the extinction profiles of the sample at the beginning of the analysis and the green lines at the end of the analysis (STEP technology - space and time resolved extinction profiles).

The “fingerprints” or transmission profiles indicate changes in the particle concentration within the analyzed samples using the STEP technology (space-time resolved extinction profiles). In the case of each biopolymer, the evolution of the transmission profiles provides the necessary information on the kinetics of concentration changes caused by phase separation. Additionally, based on the transmission level through the analyzed samples, it is possible to observe the transparency of some systems. The “fingerprints” transmission profiles for each biopolymer are presented in Figure 4. The destabilization was regarded as the structural compression of the sample and the formation of a water layer on the surface.

A structural compression was observed in the case of WP, MD, and TG. However, the destabilization of the system was the fastest for maltodextrin (MD) and wheat protein (WP). Based on the transmission profiles, the most stable samples were PP, GG, KG, INU, and PS. Gellan gum (GG), konjac gum (KG), and psyllium (PS) samples had high transparency, which was indicated by the high transmission of the near-infrared (NIR) light through the cell. The light transmission was around 40% for GG, 70% for KG, and 50% for PS. Although the KG and PS samples were stable, a movement in the particles’ concentration (including the air that might have become entrapped in the structure during the preparation process) could have caused the fluctuations observed in the fingerprints.

### 3.4. Color Parameters

The mean values of the color parameters (L*, a*, and b*) and the whiteness index for each biopolymer are presented in Table 2. Each biopolymer has a characteristic color which is visible in Figure 1 and can be proved by the values of the color parameters that were significantly different for each biopolymer. The highest value of L* parameter was recorded for INU and MD. While the highest a* value was recorded for PP and the highest b* value was in the case of PP and WP. These color parameters affected the whiteness index, which ranged from 15.35 to 91.92. It is worth mentioning that the PS and TG samples had the highest whiteness index due to being the most transparent. 

To comprehensively determine the difference between the studied biopolymers, the total color difference parameter (ΔE) was calculated (Table 3). It was found that in most of the cases, ΔE values determined between different biopolymers were higher than 5, which means that the observer notices two different colors. Nonetheless, only an experienced observer could notice the difference (1 < ∆E < 2) between INU and MD. Additionally, there is a noticeable difference in the color between PP and WP.

### 3.5. Principal Component Analysis (PCA) and Hierarchal Cluster Analysis (HCA)

**Figure 5:**
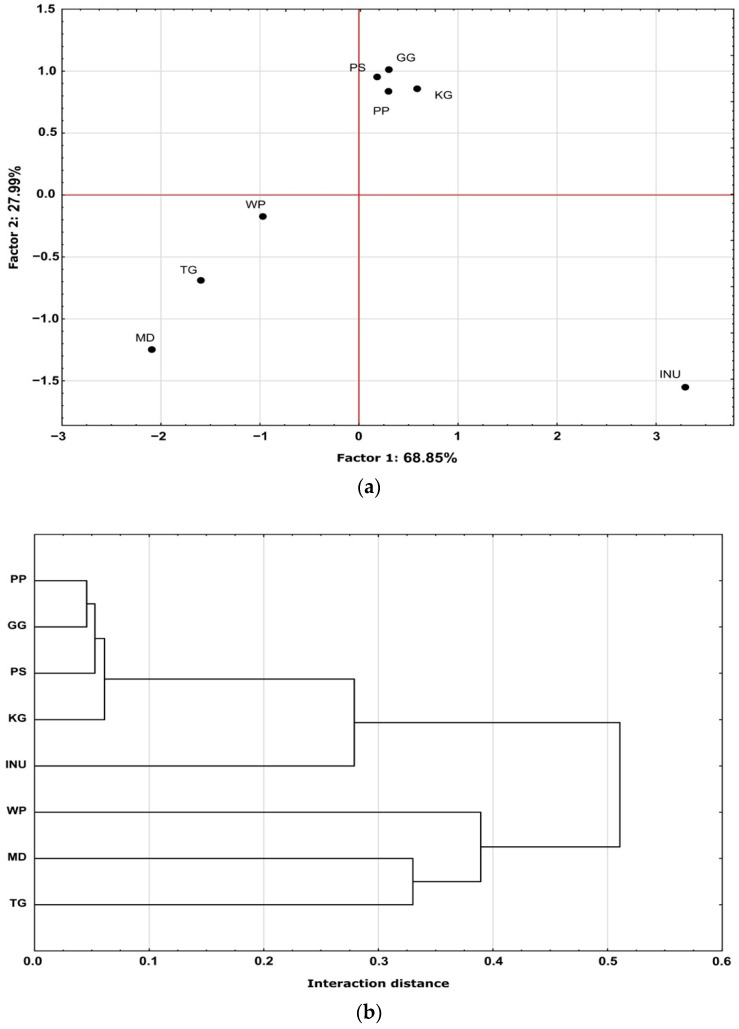
Principal component analysis PCA (**a**) and hierarchal cluster analysis HCA (**b**) of the analyzed biopolymers.

The PCA and HCA of the obtained result are presented in Figure 5. The principal component analysis (PCA) indicates the relation between the investigated parameters. Two major factors were identified: factor 1 describing 68.85% and factor 27.99% of the variance (96.84% in total). As shown in Figure 5 (PCA and HCA), the analyzed biopolymers differed significantly and could be divided into three groups. The first includes WP, MD, and TG. The second group includes PP, GG, KG, and PS. Inulin (INU) was the only biopolymer that had the largest difference in terms of both factors. However, based on HCA, INU was most similar to PP, GG, KG, and PS. PP, GG, KG, INU, and PS differed the most from WP, MD, and TG, which is also shown by HCA (the biggest distance).

## 4. Discussion

In the present study, eight biopolymers were analyzed in terms of their volumetric gelling index, microrheological properties, physical stability, and color parameters. Based on the volumetric gelling index (Figure 1), PP, GG, KG, INU, and PS formed a gel structure. This observation was confirmed by the microrheological properties of the analyzed samples. PP, GG, KG, INU, and PS exhibited a more solid-like behavior in comparison to WP, MD, and TG (Table 1). The mean square displacement profile of INU (MSD, Figure 2) was non-rectilinear and the closest to the baseline, indicating that it had the most viscoelastic properties. This observation was confirmed by INU having the highest elasticity index and macroscopic viscosity index value, which were caused by the formation of a gel structure (network). When using physical (mechanical and thermal) induction techniques, inulin can form a hydrogel with a sponge-like structure. The formation of inulin hydrogel is based on particles attraction caused by Van der Waals forces [57]. Furthermore, Beccard et al., (2019) [58] in their studies stated that inulin gelation is based on a crystallization process, which explains why (in terms of PCA and HCA, Figure 5) inulin hydrogels differed significantly from the other biopolymers. On the other hand, PP, GG, KG, and PS had significantly comparable microscopic viscosity index values, which means that the particles movement was similar in each sample. In the case of pea protein (PP), the gel structure depends on the ratio of soluble and non-soluble protein molecules that might disturb the gel structure due to the difference in the degree of cross-linking. The high elasticity of the pea protein hydrogel might suggest that a high number of soluble aggregates formed a network, leading to a highly dense structure [59]. Based on the induction technique (heat-set gelation, while pH > 6 or < 4), it is possible to obtain a fibrillar (linear) aggregates network with high elasticity [60]. Similarly, gellan gum (GG) [61], konjac gum (KG) [62], and psyllium [63] exhibit a similar ability to form a fibrillar gel network with junction zones (stabilized by hydrogen bonds, electrostatic forces, hydrophobic interactions, Van der Waals attractions, and molecular entanglement). This might explain the similarities in terms of the analyzed parameters between PP, GG, KG, and PS (Figure 5). Moreover, in the case of the solid–liquid balance (SLB), PP, GG, KG, INU, and PS had the lowest values (SLB < 0.5), which confirms the formation of a gel structure (G’ > G”)—the samples exhibited typical solid-like (elastic) behavior [64]. On the other hand, WP, MD, and TG did not form a gel structure, which was observed based on the MSD profiles (Figure 2). Although WP had a non-rectilinear profile, the results shown in Table 1 confirmed the dominance of liquid-like behavior over the solid one. However, in the studies conducted by Wang et al., (2017) [65] concerning the changes in chemical interactions and protein conformation during heat-induced wheat gluten gel formation, the authors stated that the heat treatment (above 60 °C) of a wheat protein dispersion resulted in the formation of a wheat protein gel structure. The formation of wheat protein hydrogel is related to the presence of glutenin, which after hydration is responsible for the strength and elasticity of the gel structure. However, in the case of a less flexible (brittle) gel structure, wheat protein might contain more gliadin [66]. Based on Kanyuck et al., a (2019) study [67] concerning the influence of temperature on network formation of low DE maltodextrin gels, it can be stated that high induction temperature may weaken the gel structure of maltodextrin. The temperature could be the reason for the lack of the gel structure in the analyzed maltodextrin samples. The tara gum aqueous dispersion exhibited a predominantly viscous behavior. This is in accordance with the study by Huamaní-Meléndez et al., (2021) [68] in which they stated that tara gum has thickening abilities comparable to guar and locus gum. 

The physical stability results (instability index and fingerprints, Figure 3 and Figure 4) suggest that the gel structure formed by PP, GG, KG, and INU significantly affected the stability of the samples (instability index < 0.02). PS was also stable (0.06), but this instability index value was significantly higher in comparison with PP, GG, KG, and INU. This might be due to psyllium containing husk particles (visible in Figure 1) which have sedimented during the test. The high physical stability of PP, GG, KG, INU, and PS might be due to the formation of a network that acted as a stabilizing structure when the samples were subjected to the centrifugal force during the test. Florowska et al., (2022) [19] in their studies on inulin hydrogels with the addition of sodium alginate and chitosan, also reported the high physical stability of inulin hydrogels. Furthermore, the hydrogel’s water-holding capacity is related to its physical stability [69]. This relation was stated in Qayum et al., (2021) [70] a study in which they observed that the uniform and compact structure formed by lactalbumin affects the centrifugal (physical stability) and water-holding capacity of the obtained gels. High physical stability is critical in biomedical applications, particularly in tissue engineering, when designing a scaffold using a solid free fabrication technology to ensure the preparation of a 3D matrix in the desired morphology, capable of supporting tissue growth [71,72]. However, in the case of WP, MD, and TG, the samples were highly unstable due to the absence of a gel structure. According to Feng et al., (2021) [73], the low stability of polymeric network structure might be caused by the weak interactions (or lack of interaction) between the water and the polymer, which leads to a low resistance during deformation. The hydrogel structure might be destabilized by acceleration forces or vibrations during storage. Therefore, Zhang et al., (2022) [27] in their study on thixotropic composite hydrogels based on agarose and inorganic hybrid gellants, indicated the importance of increasing the ratio of the residual gel mass by adding more agarose, to achieve improved physical stability of the analyzed hydrogel. The research conducted by Florowska et al., (2020) [54] covering the addition of selected plant-derived proteins as modifiers of inulin hydrogels properties, also confirms that the addition of a gelling biopolymer (in this case protein) resulted in a more compact hydrogel structure and higher physical stability in comparison to the control sample. 

The color of hydrogels is one of the main characteristics determining the quality of the products in which they are used, and it has a decisive influence on consumer acceptance or rejection in the case of food and cosmetic products [74]. Due to the different origins of the analyzed biopolymers, their color parameter (Table 2 and Table 3) differs significantly, which was also confirmed by the images of the obtained samples in Figure 1. In the case of all the analyzed biopolymers, the observer will notice two different colors (∆E > 5). However, in the case of pea protein (PP) and wheat protein (WP) the observer can notice a clear difference in color 3.5 < ∆E < 5, while in the case of inulin (INU) and maltodextrin (MD), only an experienced observer can notice the difference between their colors. It can be also observed that GG, KG, and PS are more transparent than the other biopolymers (although PS has a higher a* and b* parameter—more yellow tones). The color parameters of inulin hydrogels are in accordance with the available literature [19,54]. Novel edible composite films made of whey protein isolate and zein also showed similar values of the color parameters to those of the pea protein (PP) and wheat protein (WP) samples. However, in the case of gellan gum, based on Li et al., (2019) [75] a study concerning the effect of gellan gum on the functional properties of low-fat chicken meat batters, it was observed that the L* value was correlated to the gellan gum structure. The increase in the water content of the meat batters caused the disruption of the gel structure, resulting in a lower lightness. Therefore, when designing a new product, the color parameters of the hydrogel are crucial, as they can influence the overall reception of the final product.

## 5. Conclusions

Based on the achieved results, and in the investigated gelling conditions, pea protein, gellan gum, konjac gum, psyllium, and inulin had the most promising gelling ability—they were able to produce highly elastic and physically stable hydrogels. Moreover, pea protein, gellan gum, konjac gum, and psyllium hydrogels had similar structural and biomechanical properties, while inulin hydrogel had the most diverse properties. Wheat protein, maltodextrin, and tara gum were similar in terms of the analyzed properties and did not form a gel structure. Since the combination of two biopolymers might result in hydrogels characterized by a broader range of structural and biomechanical properties, and enhanced interdisciplinary, and biomedical application potential, additional studies are currently being conducted.

## Figures and Tables

**Table 1:** Comparison of the microrheological parameters of the analyzed biopolymers.

Samples	Microrheology Parameters		
	SLB [nm^−2^]	EI × 10^−2^ [nm^−2^]	MVI × 10^−4^ [nm^−2^]
PP	0.43 ^ab^ ± 0.01	2.30 ^a^ ± 0.25	53.50 ^b^ ± 0.76
WP	0.57 ^b^ ± 0.03	0.55 ^a^ ± 0.05	13.58 ^a^ ± 2.85
GG	0.39 ^a^ ± 0.02	0.19 ^a^ ± 0.02	59.98 ^b^ ± 2.20
KG	0.34 ^a^ ± 0.13	3.70 ^a^ ± 1.25	81.58 ^b^ ± 3.75
INU	0.32 ^a^ ± 0.01	31.05 ^b^ ± 3.54	590.17 ^c^ ± 20.14
MD	0.93 ^c^ ± 0.01	0.11 ^a^ ± 0.01	0.04 ^a^ ± 0.00
PS	0.39 ^a^ ± 0.04	0.22 ^a^ ± 0.05	45.62 ^b^ ± 5.21
TG	0.94 ^c^ ± 0.01	0.08 ^a^ ± 0.01	0.25 ^a^ ± 0.01

All values are mean with standard deviation (*n* = 3). According to Turkey’s test, the values followed by the same letter (a–c) do not differ significantly (*p* > 0.05).

**Table 2:** The color parameters (L*, a*, and b*) and the whiteness index of the analyzed biopolymers.

Samples	Color Parameters			
	L*	a*	b*	WI
PP	72.45 ^f^ ± 0.02	3.44 ^g^ ± 0.00	20.31 ^g^ ± 0.00	15.35 ^a^ ± 0.02
WP	68.63 ^e^ ± 0.04	1.30 ^f^ ± 0.03	20.75 ^g^ ± 0.07	22.68 ^b^ ± 0.01
GG	42.53 ^d^ ± 0.02	−1.27 ^a^ ± 0.00	−2.48 ^a^ ± 0.09	24.61 ^c^ ± 0.02
KG	22.69 ^b^ ± 1.32	−0.19 ^d^ ± 0.08	−0.58 ^c^ ± 0.37	42.46 ^d^ ± 1.32
INU	92.06 ^h^ ± 0.18	−0.77 ^b^ ± 0.02	1.23 ^e^ ± 0.15	62.37 ^e^ ± 0.15
MD	90.41 ^g^ ± 0.25	−0.44 ^c^ ± 0.02	0.73 ^d^ ± 0.10	65.60 ^f^ ± 0.25
PS	24.78 ^c^ ± 0.21	0.13 ^e^ ± 0.08	5.00 ^f^ ± 0.16	90.37 ^g^ ± 0.20
TG	15.37 ^a^ ± 0.44	−0.19 ^d^ ± 0.03	−2.00 ^b^ ± 0.10	91.92 ^h^ ± 0.44

All values are mean with standard deviation (*n* = 3). According to Turkey’s test, the values followed by the same letter (a–h) do not differ significantly (*p* > 0.05).

**Table 3:** The color difference parameter (∆E) between the analyzed biopolymers (values are mean; *n* = 3).

Samples	PP	WP	GG	KG	INU	MD	PS	TG
**TG**	61.38	57.93	27.18	7.45	76.75	75.08	11.72	-
**PS**	50.17	46.60	19.31	5.96	67.38	65.765	-	
**MD**	26.84	29.63	47.98	67.73	1.75	-		
**INU**	27.68	30.56	49.66	69.39	-			
**KG**	54.08	50.67	19.96	-				
**GG**	37.89	35.03	-					
**WP**	4.40	-						
**PP**	-							

Depending on the ΔE values the color difference between the samples can be estimated as not noticeable for the observer (0 < ∆E < 1), only experienced observer can notice the difference (1 < ∆E < 2), unexperienced observer also notices the difference (2 < ∆E < 3.5), clear difference in color is noticed (3.5 < ∆E < 5) and observer notices two different colors (5 < ∆E).
